# Ultrasound-guided venous cannulation: a model of training between medical students and emergency physicians

**DOI:** 10.1186/cc10156

**Published:** 2011-06-22

**Authors:** UAP Flato, HP Guimaraes, O Berwanguer

**Affiliations:** 1 Instituto de Ensino e Pesquisa do Hospital do Coração - Associação do Sanatório Sírio, São Paulo - SP, Brazil

## Introduction

Use of ultrasound introduced as part of intensive care therapy makes viable bedside invasive procedures and diagnosis. Due to portability, combined with team training, its use guarantees less complications related to insertion, as well as patients' safety. It also reduces severe conditions related to the catheter, such as pneumothorax among others. The aim of this study was to evaluate the accuracy related to ultrasound-guided venous catheter insertion in a low-cost famtoma among medical students of third-year graduation compared with experienced doctors and medical residents. We evaluated the success rate of insertion, the number of puncture attempts and the time related to the insertion of the needle from contact with the surface of the phantom and its correct placement in the vein.

## Methods

Study participants were 25 undergraduate students of medicine (third year) participating in the curriculum of emergency medicine and intensive care, nine medical residents (internal medicine) and nine critical care physicians. All participants had no previous experience with ultrasound-guided procedures, and medical students had no previous experience with central venous access puncture. There was a lecture prior to the study of 2 hours in ultrasound-guided venous cannulation. Evaluation of the average time between groups was performed by ANOVA using data processing in rank due to lack of homogeneity and the Tukey test for multiple comparisons. A possible relationship between the time needed until the puncture is performed and length of experience was assessed by Spearman correlation, due to lack of normality in the data.

## Results

We found a success rate of 100% in the insertion of a catheter in phantom among all participants, a longer time in the group of graduate students (Table 1), as well as the number of punctures (mean of 2).

**Table 1:** Time (seconds) to cannulation in each group

Group	*n*	Mean	Standard deviation	*P *value
Inexperienced	25	19.60	13.778	
Residents	9	12.44	5.525	0.003
Experts	8	9.88	1.553	
Total	42	16.21	11.638	

## Conclusion

The use of ultrasound-guided cannulation is a reliable method of training associated with a high of success among graduate students and experienced professionals.

